# Independent mitochondrial and nuclear exchanges arising in *Rhizophagus irregularis* crossed-isolates support the presence of a mitochondrial segregation mechanism

**DOI:** 10.1186/s12866-016-0627-5

**Published:** 2016-01-23

**Authors:** Laurence Daubois, Denis Beaudet, Mohamed Hijri, Ivan de la Providencia

**Affiliations:** Institut de Recherche en Biologie Végétale, Université de Montréal, 4101 Sherbrooke Est, Montréal, H1X 2B2 QC Canada

**Keywords:** Arbuscular mycorrhizal fungi, Mitochondrial segregation, Anastomosis, Mitochondrial nucleoids, Protein orthology, Monosporal cultures

## Abstract

**Background:**

Arbuscular mycorrhizal fungi (AMF) are members of the phylum Glomeromycota, an early divergent fungal lineage that forms symbiotic associations with the large majority of land plants. These organisms are asexual obligate biotrophs, meaning that they cannot complete their life cycle in the absence of a suitable host. These fungi can exchange genetic information through hyphal fusions (i.e. anastomosis) with genetically compatible isolates belonging to the same species. The occurrence of transient mitochondrial length-heteroplasmy through anastomosis between geographically distant *Rhizophagus irregularis* isolates was previously demonstrated in single spores resulting from crossing experiments. However, (1) the persistence of this phenomenon in monosporal culture lines from crossed parental isolates, (2) its correlation with nuclear exchanges and (3) the potential mechanisms responsible for mitochondrial inheritance are still unknown. Using the AMF model organism *R. irregularis*, we tested whether the presence of a heteroplasmic state in progeny spores was linked to the occurrence of nuclear exchanges and whether the previously observed heteroplasmic state persisted in monosporal *in vitro* crossed-culture lines. We also investigated the presence of a putative mitochondrial segregation apparatus in Glomeromycota by identifying proteins similar to those found in other fungal groups.

**Results:**

We observed the occurrence of biparental inheritance both for mitochondrial and nuclear markers tested in single spores obtained from crossed-isolates. However, only one parental mitochondrial DNA and nuclear genotype were recovered in each monosporal crossed-cultures, with an overrepresentation of certain mitochondrial haplotypes. These results strongly support the presence of a nuclear-independent mitochondrial segregation mechanism in *R. irregularis*. Furthermore, a nearly complete set of genes was identified with putative orthology to those found in other fungi and known to be associated with the mitochondrial segregation in *Saccharomyces cerevisiae* and filamentous fungi.

**Conclusions:**

Our findings suggest that mitochondrial segregation might take place either during spore formation or colony development and that it might be independent of the nuclear segregation machinery. We present the basic building blocks for a better understanding of the mitochondrial inheritance process and segregation in these important symbiotic fungi. The comprehension of these processes is of great importance since it has been shown that different segregated lines of the same isolate can have variable effects on the host plant.

**Electronic supplementary material:**

The online version of this article (doi:10.1186/s12866-016-0627-5) contains supplementary material, which is available to authorized users.

## Background

It has been widely documented that major food crops associate naturally with beneficial soil microbes, including the arbuscular mycorrhizal fungi (AMF), an ancient group of root-inhabiting fungi belonging to the phylum Glomeromycota [1]. These ecologically crucial symbionts enhance the uptake of water and nutrients of plants they colonize, especially phosphate [2], as well as protect them against pathogens [3] and play a key role in soil structure [4]. The benefits to plants provided by this association with AMF could be enhanced by the manipulation of the fungal partner genetics, as demonstrated by Angelard et al. [5] and Colard et al. [6], using the model organism *Rhizophagus irregularis*. In both studies, through the exchange of nuclear genetic information via hyphal fusion, *in vitro* crossed-culture lines and segregated culture lines were generated. This process influenced differentially the transcription of symbiosis-specific genes in rice, resulting in an increase in rice growth by a factor of five.

During the last decade numerous studies have been devoted to decipher the nuclear [7] and mitochondrial genome organization patterns [8, 9], the sexual/asexual conundrum [10–12] and nuclear segregation process in AMF [13]. These data allowed new insights into the nuclear inheritance processes and segregation mechanisms that could occur in these fungi. Despite this progress, little is known about mitochondrial inheritance in AMF. Since the mitochondrial genome encodes essential components of the cellular energy-producing apparatus, understanding mitochondrial DNA (mtDNA) organization and inheritance in AMF is of paramount importance, in order to manage more efficiently mycorrhizal associations at large scale [14]. A previous study showed that AMF mtDNAs and nuclei migrate massively into spores during their formation [15], but the fate of each parental haplotype following crosses needs to be investigated.

The publication of 14 complete AMF mtDNA [9, 16–20] in the last few years allowed demonstrating that these sequences were all homoplasmic within isolates [16, 17, 21, 22]. These studies also showed that genomes are variable in defined intergenic regions between isolates, thus offering an unparalleled opportunity to design isolate-specific markers [8, 17, 20, 23]. Recently, de la Providencia et al. [20] demonstrated length-heteroplasmy in spores formed near anastomosis regions between geographically distant *R. irregularis in vitro* isolates, using isolate-specific mitochondrial markers. However, no information is available regarding the persistence of this length-heteroplasmic state in *in vitro* monosporal culture lines established from crossed parental isolates. Such a study is needed not only to understand mtDNA inheritance processes and the persistence of mitochondrial haplotypes in stable culture lines, but also to use mtDNA as a criterion to define a reliable AMF taxonomic unit [8, 9], either at a genus, species or isolate level.

The mitochondrial inheritance process requires an active transport of organelles along the cytoskeleton and relies on membrane fission and fusion events. It plays a crucial role in the adaptation of the organism in energy requirements. Most of the molecular machinery and cellular mechanisms mediating these processes have been conserved along the fungal and animal kingdom evolution [24]. In filamentous fungi, sexual crosses lead to uniparental transmission of mitochondria [25, 26], whereas mitochondria are biparentally inherited in budding yeast [27, 28]. In yeast, microfilaments, such as actin, play an important role in the positioning and motility of mitochondria, but microtubules are the principal mitochondrial transporter in many other fungi [24]. For many years, the study of mitochondrial dynamics in fungi relied mainly on microscopic observations. However, the sequencing of fungal genomes is an important step towards a better understanding of the molecular machinery mediating mitochondrial inheritance. In that regard, the recently available *R. irregularis* genomic sequence data [7, 29] provide new tools to identify molecular components involved in mitochondrial dynamics in AMF.

No study has yet investigated potential mitochondrial segregation and/or inheritance mechanisms occurring in AMF. In the model organism *Saccharomyces cerevisiae*, mitochondrial inheritance is controlled by the mitochondrial segregation apparatus (MSA), which ensures a reliable transmission of mitochondrial organelles and their genomes to the progeny. Searches of sequence databases reveal evolutionarily conserved proteins for all known budding yeast MSA components in *Schizosaccharomyces pombe* and in filamentous fungi such as *Neurospora crassa* and *Aspergillus nidulans*, notably [30]. This suggests that the core machinery of mitochondrial inheritance has been conserved during evolution, even in organisms that use different cytoskeletal systems for organellar transport [24]. The MSA in *S. cerevisiae* is a trans-membrane proteic complex consisting of three mitochondrial membrane proteins (i.e. MMM1, MDM10 and MDM12), forming the core component of the apparatus, along with MMM2, MDM31 and MDM32, which interact with the core component (for review see [31]). This proteic complex links the mitochondrial outer membrane to the actin cytoskeleton and the mitochondrial inner membrane to mitochondrial nucleoids, which are clusters of similar mtDNAs packaged by proteins ABF2, ACO1 and ILV5 and maintained together by Holliday junctions [32–34]. However, the molecular machinery of mtDNA inheritance remains largely unknown in many other organisms [35].

In this study, we tested the hypothesis that monosporal crossed-culture lines would maintain the mitochondrial length-heteroplasmy [20] previously observed in *R. irregularis in vitro* spore progenies arising from crossed-cultures of genetically divergent isolates. In addition, we investigated nuclear inheritance following anastomosis in spore progenies and monosporal crossed-culture lines. Furthermore, we searched for the existence of a putative MSA in *Glomeromycota,* by finding the best reciprocal BLAST of yeast MSA proteins in the published genome of *R. irregularis* [7] and its transcriptome [36], and tested their orthology with all available sequences on the database.

## Results

### Germination and fungal development of monosporal culture lines

**Fig. 1:**
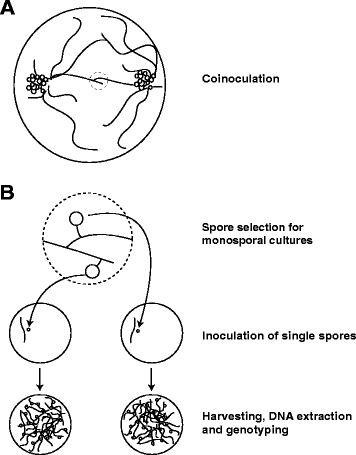
Schematic drawing of the experimental design. **a** Observation of hyphal contacts between two clusters of spores from different isolates **b** Identification of spores produced in anastomosis regions and inoculation of a single spore on a new petri dish with Ri t-DNA transformed chicory roots *i.e.* monosporal cultures. After germination and colonisation of the petri dish, spores are extracted and their DNA is used for genotyping of the monosporal culture

Cultures from each combination (DAOM-197198/DAOM-234328; DAOM-197198/DAOM-240415; DAOM-234328/DAOM-240415) were performed. After identifying interaction zones between mycelia from different isolates, which were characterized by the formation of hyphal contacts, randomly chosen spores (i.e. progenies) were harvested from the interaction zone of each combination, individually cut out from the mycelium and placed in a new Petri dish in association with Ri T-DNA transformed chicory (*Cichorium intybus*) roots as described in the methods section (Fig. 1). For each combination, approximately half of the inoculated spores germinated. The observed germination rate corresponds to rates previously observed in four *in vitro* cultured *Glomeraceae* species [15]. Following root contact and colonisation, some cultures stopped their growth, without producing any spores, and therefore could not be used in this study. The rate of mycelium development and spore production greatly varied among cultures, the combination of isolates DAOM-197198 and DAOM-240415 being the most successful, resulting in 18 colonized dishes containing between 200 and 1000 newly formed spores. The combination of isolates DAOM-197198 and DAOM-234328 produced seven colonized Petri dishes with few, newly formed spores (10–40 spores/plate). We obtained 11 poorly developed cultures from isolates combination DAOM-234328/DAOM-240415, and only nine produced sufficient amount of fungal material required for further analysis. In total, 34 monosporal crossed-cultures lines were genotyped.

### Genotyping analysis

**Fig. 2:**
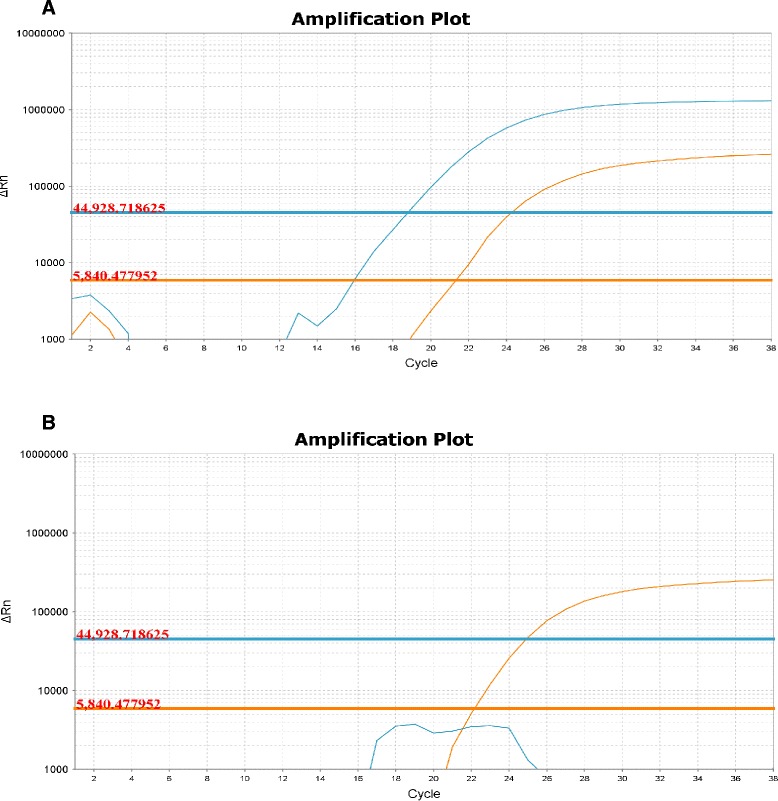
Amplification curve of qPCR assay. **a** amplification curves of a heteroplasmic spore showing two amplification curves corresponding to each parental haplotype *R. irregularis* DAOM197198 (*in orange*) and *R. irregularis* DAOM240415 (*in blue*) **b** amplification curve of a homoplasmic monosporal culture assay containing both markers but showing the presence of only one parental haplotype; *R. irregularis* DAOM197198 (*in orange*) and *R. irregularis* DAOM240415 (*in blue*)

All mitochondrial markers showed to be isolate-specific when challenged against other *Rhizophagus* isolates (Table 1). The 34 monosporal crossed-culture lines genotyped by qPCR approach presented only one parental mtDNA haplotype. In all nine progenies of the combination DAOM-197198/DAOM-234328, we only detected the DAOM-197198 haplotype, while in the combination DAOM-240415/DAOM-234328, all seven cultures exhibited the DAOM-240415 haplotype. However, among the 18 monosporal cultures of the combination DAOM-197198/DAOM-240415, two cultures showed the DAOM-197198 haplotype and the other 16 cultures were generated with the DAOM-240415 haplotype. Genotyping results are summarized in Table 2. The qPCR data are shown in the Additional file 1. Interestingly, our results showed an apparent selection and/or segregation bias towards a given haplotype in each combination. Indeed, in combinations where the DAOM-240415 haplotype was present, it seemed to be preferentially selected over the two other haplotypes. Also, the DAOM-197198 haplotype dominated when it was combined with the DAOM-234328 haplotype, the latter was not detected in any monosporal culture lines. We also corroborated the heteroplasmy detected in an earlier study by de la Providencia et al. [20], although at a lower rate, using the same genetic material from single spores, thus confirming non-self fusion between genetically-close isolates as an important mechanism shaping genetic exchange (Table 2 and Fig. 2). Two nuclear markers, BG112 and BG62, previously described and suggested for *R. irregularis* genotyping [5, 37, 38], were used to determine the nuclear inheritance in single spores [20] as well as in monosporal crossed-cultures lines. For nuclear markers tested in single spore progenies, we observed different scenarios: (1) only one parental mitochondrial haplotype, with the corresponding parental nuclear genotype (2) one parental mitochondrial haplotype and both parental nuclear genotype (3) both parental mitochondrial haplotypes and only one parental nuclear genotype. Interestingly, out of 12 successfully analysed single spores, four showed the presence of both parental markers. Missing data correspond to incomplete PCR reactions due low amount of genomic DNA remaining from previous experiments published by de la Providencia [20]. In crossed-culture lines, no biparental nuclear inheritance was detected (see Additional file 2).

**Table 1:** Specificity test of qPCR markers. The marker for each isolate is, at least, specific to the other two isolates used in this study

		*R. irregularis* DAOM								*G. cerebriforme DAOM227022*	Water
	Marker\DNA	197198	240415	234328	197198/240415	240415/234328	197198/234328	242422	234179		
Singleplex	197198	X (19)	-	-	X (19)	-	X (19)	X (19)	-	-	-
	240415	-	X (21)	-	X (21)	X (21)	-	-	-	-	-
	234328	-		X (26)	-	X (26)	X (27)	-	X (27)	-	-
Multiplex	197198	X (19)	-	-	X (19)	-	X (19)	-	-	-	-
	240415	-	X (21)	-	X (21)	X (21)	-	-	-	-	-
	197198	X (19)	-	-	X (19)	-	X (19)	X (19)	-	-	-
	234328	-	-	X (26)	-	X (26)	X (27)	-	X (27)	-	-

Adequate controls were carried, in triplicate, both in singleplex and multiplex. Multiplex reaction could not be performed with the combination of 240415 and 234328, because of limitations of the calibrated fluorophores and filter-limitations of the instrument. For each positive assay, in parenthesis is the mean ct value from triplicates with 2 ng of DNA per essay

**Table 2:** Characterization of monosporal cultures from crossing experiments with three different *Rhizophagus irregularis* isolates and single spores from de la Providencia et al. [20]

Combinations	Inoculated monosporal cultures	Colonized monosporal cultures	Spores per culture	* R. irregularis* haplotype		
				DAOM197198	DAOM240415	DAOM234328
* R. irregularis* 197198/240415	50	18	100–1000	2	16	NA
* R. irregularis* 197198/234328	50	9	50–300	9	NA	0
* R. irregularis* 240415/234328	50	7	30–100	NA	7	0
de la Providencia et al. (2013) [20]						
Combinations	Heteroplasmic spores			Homoplasmic spores		
	Previous markers	New markers		Previous markers		New markers
* R. irregularis* 197198/240415	9	3		1		7
* R. irregularis* 197198/234328	5	0		5		10
* R. irregularis* 240415/234328	3	1		7		9

### Mitochondrial segregation machinery and nucleoid genes orthology in *R. irregularis*

**Fig. 3:**
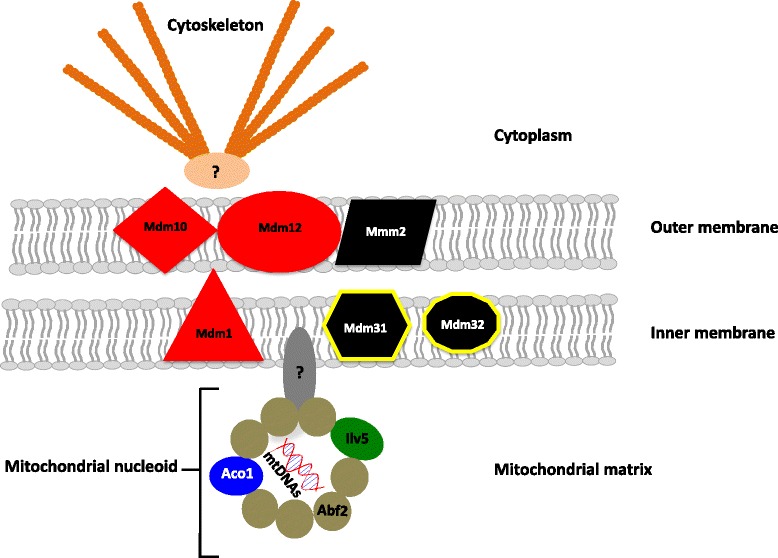
Representation of the putative mitochondrial segregation apparatus and nucleoid structure in *Rhizophagus irregularis.* Based on protein orthology analysis with *S. cerevisiae* and other filamentous fungi. The core components of this machinery consist of a complex of three mitochondrial membrane proteins, which are MMM1, MDM10 and MDM12 (*colored in black*). Three proteins are thought to interact with the main complex, MMM2, MDM31 and MDM32 (*colored in red*). The proteins responsible for mtDNA packaging and nucleoid formation are ABF2, ACO1 and ILV5 (*colored in brown*, *blue and green*, respectively). Unknown proteins are thought to link the outer membrane complex to the cytoskeleton (*colored in orange*) and the nucleoids to the inner membrane by interacting with MMM1. The yellow outlining shows proteins for which no putative orthologs were found in *R. irregularis* (Table 3)

The genome of *R. irregularis* DAOM-197198 [7] possesses a nearly full set of genes that exhibited a high degree of similarity to genes coding for the core structure of the MSA and involved in the formation of nucleoids in *S. cerevisiae* and other filamentous fungi (Table 3 and Fig. 3). Orthologous candidates in *R. irregularis* corresponded to the best reciprocal blast hit with the *S. cerevisiae* MSA protein (Table 3). To confirm orthology, phylogenetic trees were constructed using all known orthologous genes in fungi and other organisms. All seven phylogenies performed with clusters of orthologous genes (COGs) supported the idea that *R. irregularis* proteins are putative orthologs of fungal segregation apparatus and nucleoid proteins (see Additional file 3). It is noteworthy that in most cases the *R. irregularis* proteins come out basal in our analyses with low bootstrap support, probably given the lack of sequences availability belonging to lower fungal taxa. The presence of one *S. cerevisiae* paralog was observed in the ACO1 phylogeny, as well as two yeast homologs and one paralog in ABF2 COG phylogeny. However, in both cases, the *R. irregularis* protein grouped with the expected *S. cerevisiae* protein, playing a role in the mitochondrial segregation processes. We did not find any paralogs in *R. irregularis*. Only two genes were missing in *R. irregularis*, *i.e. mdm31* and *mdm32*. However, their role in the mitochondrial inheritance process remains unclear in yeast. They are speculated to play an interactive role with the segregation apparatus core components [39]. Furthermore, for these two proteins, the search for COGs recovered only a few yeast sequences, which leads us to believe they might not be necessary to the segregation process in filamentous fungi. All putative orthologs found in *R. irregularis* are likely functional since they are also found in expressed sequence tags (ESTs) [36].

**Table 3:** Evidence for *Rhizophagus irregularis* protein orthology with the *Saccharomyces cerevisiae* mitochondrial segregation and nucleoid proteic machinery

Protein^a^	Primary function^a^	Best reciprocal BLAST	E-value	Similarity %	Phylogenetic orthologs^b^	mRNA expression^c^
MDM10	Core components of the mt segregation apparatus	●	1e-32	44	●	●
U9UEK3						
MDM12		●	2e-15	52	●	●
U9UFF5						
MMM1		●	3e-53	55	●	●
U9U1X0						
MMM2	Putative interaction with the mt segregation apparatus	●	5e-11	53	●	●
U9TTI3						
MDM31		○	○	○	○	○
MDM32		○	○	○	○	○
ILV5	Biosynthesis of Val, Ile and Leu	●	1e-158	76	●	●
U9UJR1						
ACO1	Citric acid cycle	●	0.0	84	●	●
U9UI83						
ABF2	mtDNA packaging	±^d^	1e-14	48	●	●
U9UF16						

^**a**^Described proteins and known functions in *Saccharomyces cerevisiae* (Chen et al. [55]), along with the accession number of the *R. irregularis* ortholog gene candidates

^**b**^As shown in Additional file 3

^**c**^Based on the *R. irregularis* transcriptome data (Tisserant et al. [36])

^**d**^Two proteins in *R. irregularis* and *S. cerevisiae* showed close similarity to each other because they share the same high mobility HMG-BOX proteic domains

## Discussion

### Homoplasmy rather than length-heteroplasmy in monosporal cultures lines from crossed-cultures

Previous results have shown that biparental mtDNA inheritance leading to a heteroplasmic state occurred in the spore progeny from crossed-cultures of divergent *R. irregularis* isolates, however heteroplasmy was not detected in germinated spores [20]. Using TaqMan markers developed in the present study, we did not detect heteroplasmy in monosporal cultures from crossed parental isolates but confirmed the heteroplasmic status of the crossed-cultures spores observed in de la Providencia et al. [20], although at a lower rate. Several factors might explain why we obtained dissimilar results with markers designed in different intergenic regions. The isolate-specific mitochondrial markers are designed in highly variable mobile elements rich regions, which have been shown to be recombination prone [8] and might compromise their specificity. Also, the use of the whole genome amplification (WGA) technique [20] can introduce significant bias regarding the insertions of SNPs or the formation of chimeras [40, 41]. The latter could induce an overestimation of the presence of a given haplotype in a sample, especially since the occurrence of allelic drop-out and preferential amplification is well documented in single cell analysis using a PCR based approach [42]. For these reasons, TaqMan qPCR assay is more reliable to assess mtDNA inheritance, given the nature of the highly variable/dynamic regions in which AMF isolate specific markers are designed.

Earlier studies have demonstrated that genetic exchanges occur via vegetative hyphal fusion (i.e. anastomosis), resulting in nuclei coexisting in a common cytoplasm, altering both the plant and fungal phenotypes [5, 6, 13, 20, 37, 43]. Based on the formation of anastomosis between genetically-close *R. irregularis* isolates, several studies have shown that heterogeneous populations of nuclei [5, 6] and mtDNA [20] are randomly inherited at different frequencies (i.e. genetic drift and/or segregation) [44] into the progeny [15]. These findings support the paradigm that based on the coenocytic nature of the AMF fungal mycelium, nuclei, mitochondria and other organelles can migrate between close or distant regions of genetically-close fungal colonies and form the so-called common mycorrhizal networks (CMN) [45]. However, studies on nuclear dynamics along the symbiotic extraradical mycelium challenged this point of view and brought a somewhat discordant note to the supposed continuous stream of mycelial cytoplasm/protoplasm [46]. Using *in vivo* two-photon microscopy techniques, these authors revealed a patchy distribution of nuclei throughout the mycelia and also demonstrated that nuclear flow occurs in pulses, being independent from the cytoplasmic streaming. These studies suggest that mitochondria, like nuclei, might not be equally distributed along hyphae. Therefore, through anastomosis originating from different individuals, the inheritance of mitochondria into the spore progeny could be strongly related to the number, type and frequency of the organelle at the interaction zone (i.e. zone of the mycelium where genetically-close individuals fuse and exchange nutrients and genetic material). The latter could explain the lack of detection of a heteroplasmic state in the progeny spore used to start the monosporal culture. In the event that one haplotype would be crucially underrepresented and therefore the heteroplasmy undetectable by a qPCR assay, such a small amount of a mitochondrial haplotype could easily be lost through stochastic drift in subsequent subcultures, and consequently homoplasmy would persist at the vegetative phase in these fungi. The dominance of some haplotypes we observed would still imply a selection and/or segregation bias of one haplotype over the other.

Together with our observations of a dominant homoplasmic state in the monosporal crossed-culture lines, these studies strongly support a plausible mechanism of mtDNA segregation. This in turn offers an explanation for the low frequency, revised in this study (Table 2), of heteroplasmic spores resulting from crossed, geographically and genetically distant isolates [20] and the non-detection of heteroplasmic monosporal crossed-cultures lines arising from these spores. Moreover, results obtained with nuclear markers show that spores can have both parental nuclear markers, whereas only one mitochondrial parental haplotype. This suggests that both nuclear and mitochondrial inheritance are governed by apparently independent segregation mechanisms.

### Evidence for a mitochondrial segregation mechanism in *Rhizophagus irregularis*

Although orthology analyses were not entirely conclusive given the basal positioning of the *R. irregularis* sequences in most cases, the identification of putative orthologs in *R. irregularis* genome and transcriptome to the MSA and nucleoid proteins found in other fungi offers a hypothesis to explain the mitochondrial segregation we observed in this study. The MMM1-like, MDM10-like and MDM12-like proteins are an integral part of the mitochondrial membranes and constitute the core components of the MSA. This proteic complex connects the mitochondrial outer membrane to the cytoskeleton in other fungi [47, 48], while MMM1 is thought to link mitochondrial nucleoids to the inner membrane of the mitochondria [49]. Proteins involved in the MSA have not been reported to play a role in other molecular pathways in *S. cerevisiae* or filamentous fungi and are well-conserved through fungal evolution [24]. If indeed mitochondria were linked to the cytoskeleton (i.e. either actin or microtubules), along with their mtDNA bound to the inner membrane, their movement through the cytoplasm would not be as free as actually alleged. This hypothesis could potentially explain why the fungal protoplasm moves slower after anastomosis between two divergent isolates as observed earlier [20]. Also, three proteins of the mitochondrial membrane are also proposed to interact with the core components of the MSA, which are MDM31, MDM32 and MMM2. They are thought to play a role in nucleoid stability, mitochondrial morphology and distribution in yeast [39, 50]. However, we only found a putative ortholog for the MMM2 protein, which might suggest that other proteins could play the same function as MDM31 and MDM32 or that they are not essential in the AMF mitochondrial segregation pathway. Given the phylogenetic divergences between Glomeromycota and higher fungi (i.e. Ascomycota and Basidiomycota) further phylogenetic analysis will need to include putative orthologous and paralogous genes belonging to basal fungi. Currently ongoing sequencing efforts, such as the JGI 1000 fungal genome projects, will greatly help to unravel these evolutionary relationships [51].

Furthermore, we identified putative orthologs for the three proteic constituents of mt-nucleoids in AMF. The ABF2 protein is the core packaging element of mt-nucleoids in yeast. It is a non-histone DNA binding high mobility group (HMG) protein, which shows homology to nuclear chromatin proteins [52]. This homology explains the difficulty of obtaining the best reciprocal blast (Table 3). The COG used to perform the ABF2 phylogeny (see Additional file 3) included other proteins that were homologs, and also paralogs, since *S. cerevisiae* underwent an ancient genome duplication [53]. The two other essential components of mt-nucleoid are the bi-functional proteins ACO1 and ILV5 [54, 55]. They are both highly conserved in AMF, probably due to their metabolic role in the citric acid cycle and amino acid biosynthesis, respectively [31, 55, 56]. Their expression is metabolically regulated and they play a role in packaging mtDNA into favourable conformations, while protecting DNA against oxidative stress. The detection in *R. irregularis* genome and transcriptome of well conserved putatively orthologous proteins involved exclusively in the MSA, suggest that they are functional and that they might also be involved in a similar process in AMF. The existence of mt-nucleoids in AMF is important because it accelerates the rate at which mtDNA would segregate, since it is directly correlated to the effective population size [57]. Nucleoids were also shown to create a genetic bottleneck and to be responsible for the rapid mitochondrial segregation observed in yeast [58]. Interspecific differences in the morphology, size and distribution of mitochondrial nucleoids have been visualized using different microscopic methods in yeast [59, 60]. To further investigate these findings in *Glomeromycota*, it would be interesting to perform immunofluorescent experiments targeting AMF putative nucleoid proteins, since green fluorescent protein (GFP) tagging has been shown to be transient and render unstable transformants in these multinucleated coenocytic organisms [61].

## Conclusions

Although mitochondrial homoplasmy seems to be the rule rather than the exception in monosporal progeny originating from *in vitro* crossed-cultures, the coexistence of numerous mtDNA haplotypes in the same cytoplasm and the occurrence of homologous mitochondrial recombination might be common mechanisms in natural populations. Moreover, the observation that mitochondrial and nuclear exchanges are not necessarily correlated suggests the presence of independent mitochondrial and nuclear segregation mechanisms. This supports the presence of a mitochondrial segregation apparatus in *R. irregularis* and provides a better understanding of the mitochondrial inheritance process and segregation in these fungi. Much remains to be learned about how proteins potentially implicated in this segregation mechanism interact with other elements and influence both mtDNA and nuclear inheritance mechanisms in AMF. It would be interesting to study the dynamics of genetic exchanges in natural and/or disturbed environments in order to unravel the underlying segregation processes to better understand (1) further application of mitochondrial markers in population genetics studies and (2) their effects on both the AMF and the host plant fitness.

## Methods

### Growth conditions and maintenance of fungal cultures and roots

Monoxenically produced spores of *Rhizophagus irregularis* isolates DAOM-197198 (Pont-Rouge, Quebec, Canada), DAOM-234328 (Finland) and DAOM-240415 (Dufrost, Manitoba, Canada) were provided by the DAOM collection (Ottawa, Ontario, Canada). These three isolates were selected because of their different geographical origins and because their mitochondrial genomes have been fully sequenced [18, 20]. Spores were subcultured in association with Ri T-DNA transformed chicory (*Cichorium intybus*) roots on a modified minimal (MM) medium [62] solidified with 0.4 % (w/v) gellan gum (Sigma). Cultures of the three *R. irregularis* isolates were incubated in the dark in an inverted position at 25 °C. Several thousand spores and extraradical mycelia were obtained over a period of 12 weeks. Ri T-DNA transformed chicory roots were routinely propagated by placing actively growing root apexes on MM medium with subsequent incubation at 25 °C in the dark.

### Crossed cultures and monosporal culture lines

Twenty crossed cultures from each combination (DAOM-197198/DAOM-234328; DAOM-197198/DAOM-240415; DAOM-234328/DAOM-240415) were performed by inoculating a hundred spores in close vicinity of Ri T-DNA transformed chicory roots, opposing each other at the extreme side of a Petri plate (Fig. 1). Both colonies were checked weekly and their growth was traced in order to identify interaction zones between mycelia from different isolates, which were characterized by the formation of hyphal contacts. Subsequently, these contacts were checked under a Discovery V12 stereomicroscope (Carl Zeiss, Canada) at magnifications of 6.7–40×. Bright-field microscopy (Axio Imager M1, Carl Zeiss) was also used to observe details of the hyphal interactions at higher magnifications.

After 15 weeks of growth, randomly chosen spores (i.e. progenies) were harvested from the interaction zone of each combination, individually cut out from the mycelium and placed in a new Petri plate (90 mm) containing MM medium in the close vicinity of a Ri-T transformed chicory root. For each combination, 50 replicates, consisting of one single spore associated with a chicory root were prepared. Each plate was checked weekly for germination, root colonization and colony development over the next 11 weeks.

### DNA extraction

Spores and hyphae were harvested by dissolving the gellan gum matrix in which cultures were grown in a solution containing 0.0083 M sodium citrate and 0.0017 M citric acid. Extracted fungal material was observed under a binocular microscope in order to detect and remove any root contaminants. Spores and hyphae were gently crushed in a 1.5 ml microtube using a sterilized pestle. DNA was extracted using the DNeasy Plant Mini kit (Qiagen, Toronto, ON), according to the manufacturer’s instructions.

### Mitochondrial marker development, genotyping by real-time PCR and sequencing of progeny spores

In order to efficiently detect low copy numbers of a given mitochondrial haplotype in each monosporal culture line, TaqMan isolate-specific markers were developed for each parental isolate and were used to genotype monosporal crossed culture lines (Table 1 and see Additional files 1 and 4). Genotyping of monosporal cultures lines resulting from each crossing experiment were performed in three replicates with approximately 2.5 ng of DNA per replicate. Reactions were carried in 20 μl, using iTaq™ Universal Probes Supermix (Bio-Rad, Canada) with final primers concentration at 0.5 μM and final probes concentrations at 0.1 μM. Reactions were performed using 5′FAM, 5′VIC and 5′NED dyes and their corresponding quencher. However, due to limited availability of calibrated fluorophores and filter limitations of the instrument, we could only perform multiplex qPCR for combinations DAOM-197198/DAOM-240415 and DAOM-197198/DAOM-234328 and perform singleplex qPCR reactions for the combination DAOM-240415/DAOM234328. Real time PCR assays were performed on a ViiA™ 7 Real-Time PCR System (LifeTechnologies, Canada). PCR amplicons were visualized on a 2.5 % agarose gel stained with GelRed (Invitrogen, Canada). Successful PCR amplicons were sequenced according to the conventional Sanger technique at the Genome Quebec Innovation Center (Montreal, QC). As a control for marker specificity, the new TaqMan markers developed were challenged against *R. irregularis* (DAOM 242422 and DAOM 234179) and *R. clarus* (MUCL 46238). In addition, these markers were also tested in the spore progeny issued from the previous study of de la Providencia et al. [20], in order to confirm these earlier results.

### Sequence-based nuclear markers and genotyping

In order to assess the nuclear inheritance in single spores and monosporal cultures both arising from crossed-isolates, two previously designed and used nuclear markers were tested, locus BG112 [5, 38] and locus BG62 [37] (see Additional file 4). PCR reactions were carried in 25 μl, using Taq DNA Polymerase (Quiagen, Canada) with final primers concentration at 0.5 μM, dNTP concentration at 0.2 mM and approximately 2.5 ng of DNA. PCR products were separated in a 20 cm long, 7 % acrylamide gel using DCode Universal Mutation Detection System (Bio-Rad, Canada). Gels were run at 100 vts, 18 h for marker BG112 and 15 h for marker BG62, then stained in a SybrSafe bath for 40 min and visualized in a Gel Doc XR System (Bio-Rad, Canada) (see Additional file 5).

### Protein orthology and phylogenetic analysis

Each amino acid sequences of the *Saccharomyces cerevisiae* MSA and/or protein involved in nucleoid formation (i.e. MMM1, MDM10, MDM12, MMM2, MDM31, MDM32, ABF2, ACO1 and ILV5) were searched across the *R. irregularis* DAOM-197198 genome assembly [7] and transcriptome [36], using TBLASTN. Orthologous candidates in *R. irregularis* genome were identified using the best-reciprocal BLAST hit to these proteins. Seven putative orthologous sequences were retrieved in *R. irregularis;* Uniprot accession numbers U9U1X0, U9UEK3, U9UFF5, U9TTI3, U9UF16, U9UI83 and U9UJR1, respectively. Furthermore, clusters of orthologous genes (COGs), gathering sequences from numerous organisms, were determined using STRING version 9.05 [63] for each protein candidate. The resulting COGs were aligned using COBALT version 2.01 [64]. Phylogenetic analyses and finding the best evolutionary model was done using the integrative software TOPALI version 2.5 [65]. For each protein candidate, maximum likelihood phylogenetic analyses were done with the closest orthologs found in fungi. Phylogenies were performed accordingly to its predicted model: the MMM1 protein phylogeny was done using the JTT + I + G model, MMM2 with JTT + I + G, MDM12 with JTT + G, MDM10 with WAG + I + G, ACO1 with WAG + I + G**,** ABF2 with WAG + I + G, and finally the ILV5 phylogeny was performed using the WAG + G model. The robustness of internal branches was evaluated based on 1000 bootstrap replicates (<60 % cut-off). Tree figures were completed using TreeGraph version 2.0.47 [66].

## Availability of supporting data

*R. irregularis* DAOM-197198 whole genome sequencing project database have been deposited and are available at DNA Data Bank of Japan/European Molecular Biology Laboratory/GenBank (accession no. AUPC00000000 or http://www.ncbi.nlm.nih.gov/nuccore/AUPC00000000). *R. irregularis* DAOM197198 transcriptomic data were retrieved from the INRA GlomusDB website (http://mycor.nancy.inra.fr/IMGC/GlomusGenome/index3.html). *R. irregularis* seven putative orthologous sequences are available on Uniprot (accession number U9U1X0, U9UEK3, U9UFF5, U9TTI3, U9UF16, U9UI83 and U9UJR1). Proteic sequences are also provided in fasta format in Additional file 6. Other supporting data are presented as additional files. Alignments and phylogenetic trees files are available upon request.

## Additional files

Additional file 1:
**Table showing qPCR data (ct values) of mitochondrial genotyping.** (XLSX 45 kb) Additional file 2:
**Table showing genotyping of single spores and monosporal cultures arising from crossed isolates.** (XLSX 42 kb) Additional file 3:
**Maximum likelihood phylogenetic trees based on seven proteins implicated in the mitochondrial segregation process and nucleoid formation in** ***Saccharomyces cerevisiae*** **, along with their closest orthologs found in fungi and other organisms.** Each phylogeny was performed accordingly to its predicted model: the MMM1 protein phylogeny was done using the JTT + I + G model (a), MMM2 with JTT + I + G (b), MDM12 with JTT + G (c), MDM10 with WAG + I + G (d), ACO1 with WAG + I + G (e), ABF2 with WAG + I + G (f), and finally the ILV5 phylogeny was performed using the WAG + G model (g). Numbers on branches correspond to bootstrap support values (<60 % cut-off) on 1000 replicates. The Ascomycota are in red, the Basidiomycota are in orange, while the *Rhizophagus irregularis* sequences are in blue. (DOCX 1439 kb) Additional file 4:
**Table showing** **isolate-specific** **primers used to discriminate the three** ***Rhizophagus irregularis*** **isolates.** (DOCX 76 kb) Additional file 5:
**Polyacrylamid gel electrophoresis (PAGE) on single spores.** Lanes 1–2 and 5–6 represents *R. irregularis* DAOM197198 and DAOM234328 parental marker BG112 and BG62. Lanes 3–4 and 7–8 shows markers BG112 (3–4) and BG62 (7–8) assays on two single spores containing only the mitochondrial haplotype of *R. irregularis* DAOM197198. (PNG 2768 kb) Additional file 6:
***R. irregularis*** **DAOM197198 sequences.** This file contains proteic sequences (fasta format) of seven putative orthologous sequences, also available on Uniprot, accession numbers U9U1X0, U9UEK3, U9UFF5, U9TTI3, U9UF16, U9UI83 and U9UJR1. (DOCX 156 kb)

## Abbreviations

ABF: ARS binding factor
ACO: aconitase
AMF: arbuscular mycorrhizal fungi
BLAST: basic local alignment search tool
CMN: common mycorrhizal networks
COGs: cluster of orthologous genes
DAOM: Department of Agriculture, Ottawa, Mycology
DNA: deoxyribonucleic acid
ESTs: expressed sequence tags
HMG: high mobility group
ILV: isoleucine-plus-valine requiring
MDM: maintenance distribution and morphology
MM: modified minimal
MMM: mitochondrial of mitochondrial morphology
MSA: mitochondrial segregation apparatus
mtDNA: mitochondrial DNA
MUCL: Mycothèque de l’Université catholique de Louvain
PAGE: polyacrylamide gel electrophoresis
PCR: polymerase chain reaction
qPCR: quantitative polymerase chain reaction
Ri T-DNA: transferred DNA from the Ri plasmid of *Agrobacterium rhizogenes*
SNPs: single nucleotide polymorphisms
WGA: whole genome amplification
