# Supplementary material for: Independent mitochondrial and nuclear exchanges arising in Rhizophagus irregularis crossed-isolates support the presence of a mitochondrial segregation mechanism
Source: BMC Microbiol. 2016 Jan 23;16:11. doi: 10.1186/s12866-016-0627-5 (PMC4724407; doi:10.1186/s12866-016-0627-5)
Supplement: Additional file 3: — Maximum likelihood phylogenetic trees based on seven proteins implicated in the mitochondrial segregation process and nucleoid formation in Saccharomyces cerevisiae , along with their closest orthologs found in fungi and other organisms. Each phylogeny was performed accordingly to its predicted model: the MMM1 protein phylogeny was done using the JTT + I + G model (a), MMM2 with JTT + I + G (b), MDM12 with JTT + G (c), MDM10 with WAG + I + G (d), ACO1 with WAG + I + G (e), ABF2 with WAG + I + G (f), and finally the ILV5 phylogeny was performed using the WAG + G model (g). Numbers on branches correspond to bootstrap support values (<60 % cut-off) on 1000 replicates. The Ascomycota are in red, the Basidiomycota are in orange, while the Rhizophagus irregularis sequences are in blue. (DOCX 1439 kb) [file 12866_2016_627_MOESM3_ESM.docx]

**A - MMM1 protein**

**B - MMM2 protein**

**C - MDM12 protein**

**D - MDM10 protein**

**E – ACO1 protein**

**F – ABF2 protein**

**G – ILV5 protein**
