# Supplementary material for: Independent mitochondrial and nuclear exchanges arising in Rhizophagus irregularis crossed-isolates support the presence of a mitochondrial segregation mechanism
Source: BMC Microbiol. 2016 Jan 23;16:11. doi: 10.1186/s12866-016-0627-5 (PMC4724407; doi:10.1186/s12866-016-0627-5)
Supplement: Additional file 4: — Table showing isolate-specific primers used to discriminate the three Rhizophagus irregularis isolates. (DOCX 76 kb) [file 12866_2016_627_MOESM4_ESM.docx]

Supplementary Table 1.

| **Mitochondrial Primer** | **Primer sequence**  **(5’ - 3’)** | **Probe sequence (5’ – 3’)** | **Amplicon size (bp)** | **Position of primers in mtDNA** | | |
| --- | --- | --- | --- | --- | --- | --- |
|  |  |  |  | *R. irregularis* DAOM197198 | *R. irregularis* DAOM240415 | *R. irregularis* DAOM234328 |
| 197198F  197198R | GCTCATTCTTGAGACTCTGGAAC  CTCCTTAGCTAGTTCCTCAGTGGT | CTGCCCCTGCAACTCCCGTA | 187 | 68559-  68746 |  |  |
| 240415F  249415R | TTCCTAGGGTTGCAGAATCGATC  AGAGATTTGTAGATTCTTTACCAGGA | TCCTTGAATAGGCCGTAACGGCA | 199 |  | 68960-  69160 |  |
| 234328F  234328R | GCTAATTTCCCTTTGTATCACTTGA  GTATCCATTACAATCGTCCATGGTG | AGAAGCAAAGGAGAAAGGGCTTGCTT | 191 |  |  | 62989-  63180 |

| **Nuclear Primer** | **Primer sequence**  **(5’-3’)** | **Original Publication** |
| --- | --- | --- |
| Bg112 - up  Bg112 – down | AAGGTCATATCAAATTCTCCGATCC  GAAACTGGGAAGTCAGCTCTTGT | Angelard et al, 2010 |
| Bg62F  Bg62R | CGTCAGTAAACTTGATGTGATAAAAATGA  GCCACTTTGGACACATAGAACTAGC | Croll et al, 2009 |
