# Supplementary figures and images for: Independent mitochondrial and nuclear exchanges arising in Rhizophagus irregularis crossed-isolates support the presence of a mitochondrial segregation mechanism
Source: BMC Microbiol. 2016 Jan 23;16:11. doi: 10.1186/s12866-016-0627-5 (PMC4724407; doi:10.1186/s12866-016-0627-5)

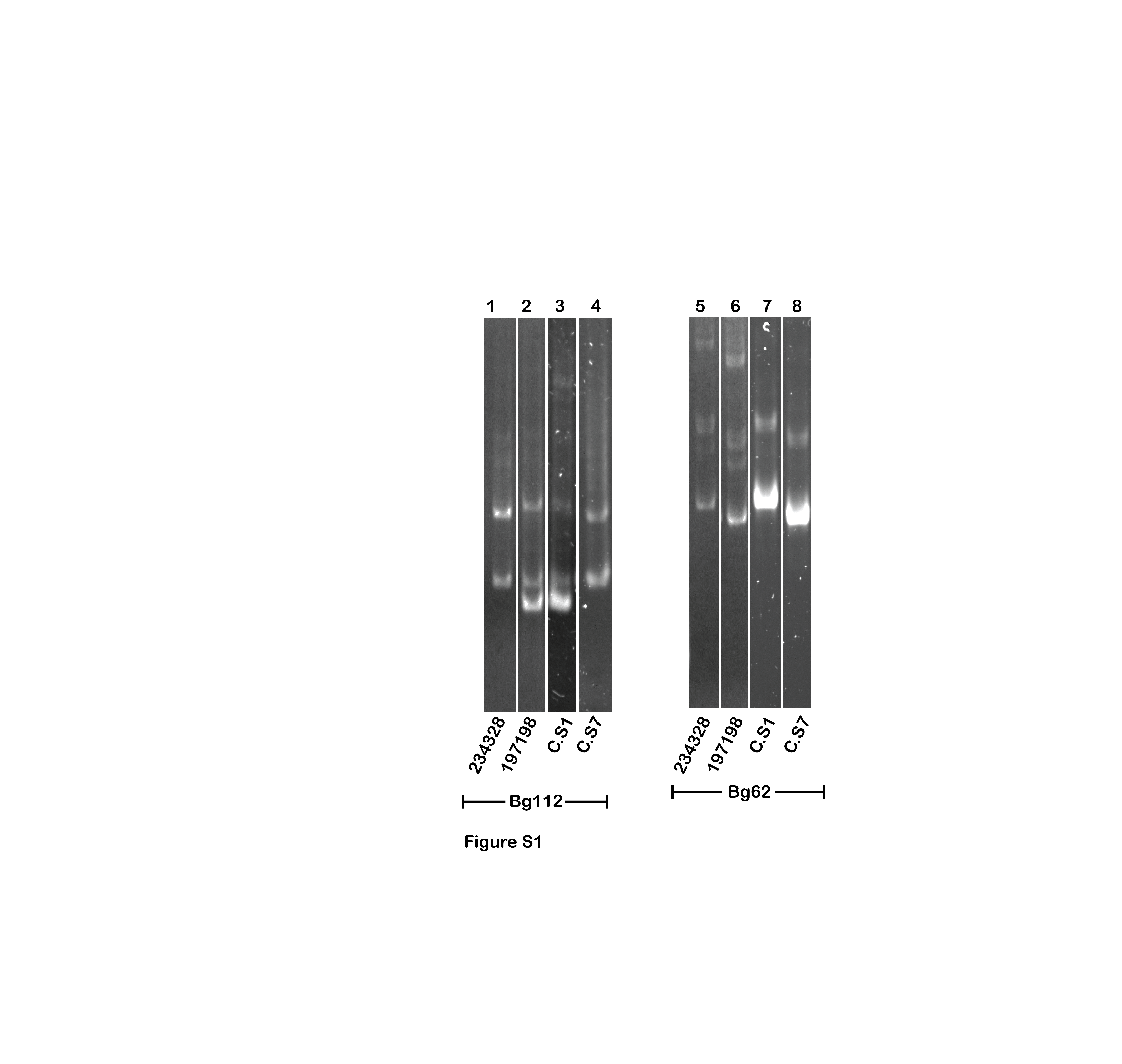

Supplement: Additional file 5: — Polyacrylamid gel electrophoresis (PAGE) on single spores. Lanes 1–2 and 5–6 represents R. irregularis DAOM197198 and DAOM234328 parental marker BG112 and BG62. Lanes 3–4 and 7–8 shows markers BG112 (3–4) and BG62 (7–8) assays on two single spores containing only the mitochondrial haplotype of R. irregularis DAOM197198. (PNG 2768 kb) [file 12866_2016_627_MOESM5_ESM.png]
