# Supplementary material for: Independent mitochondrial and nuclear exchanges arising in Rhizophagus irregularis crossed-isolates support the presence of a mitochondrial segregation mechanism
Source: BMC Microbiol. 2016 Jan 23;16:11. doi: 10.1186/s12866-016-0627-5 (PMC4724407; doi:10.1186/s12866-016-0627-5)
Supplement: Additional file 6: — R. irregularis DAOM197198 sequences. This file contains proteic sequences (fasta format) of seven putative orthologous sequences, also available on Uniprot, accession numbers U9U1X0, U9UEK3, U9UFF5, U9TTI3, U9UF16, U9UI83 and U9UJR1. (DOCX 156 kb) [file 12866_2016_627_MOESM6_ESM.docx]

>tr|U9U1X0|U9U1X0_RHIID Maintenance of mitochondrial morphology protein 1 OS=Rhizophagus irregularis (strain DAOM 181602 / DAOM 197198 / MUCL 43194) GN=MMM1 PE=3 SV=1

MSCAQSISPEVQKYLDQVVSKLPESNWSFMQGFFLGQLTVVILVLAFIKYMLLEDVKKTNLKRPLPISTPLNTKTASSSPASIILSKTLYDPASPESTAWLNVLFAQTIYQYRNDAKTDNRLVHIVDKILNSGVRPNFVGPIEVTRLDIGEEFPIFKNARIRPADALGKMRVEVDCDYSDHITLGIDTQILLNWPKPKIAVLPISLVISVIKFSATITLEIVTSPESSYILVSVLPDFILEFNVQSLIGSRSKLEDVPKITHIIISKLRNAFCENFVYPNFKKIKMPDLWSPNKNPESSDENNLQQNSYGEEINDTTKTPSTLTEGLRQRKNVIKDYPLVQDAVFLAHDVQNTTQAQVSSFN

>tr|U9UEK3|U9UEK3_RHIID Mitochondrial distribution and morphology protein 10 OS=Rhizophagus irregularis (strain DAOM 181602 / DAOM 197198 / MUCL 43194) GN=MDM10 PE=3 SV=1

MHDFMDYCLRTYFKTIGWNDDNQYSNLCSASRAILDFQTPRGLTFAISKIQFPLLKASYSMNISPILNGSLGYLFTSRPLKVDPSEHVNFIEMIDRFHINYVPKNYLHGNFDNSDNSKIKDYLLYGRLFVPTGRLEAIYSRKFSQWMQCVVTAVSDPRSKAASHVTTELQYDVGQWCTELSYTTDGELFGVRGLYNFTNYNQEEISTNSNNEESIVQNKMKKKVKDIETPLVERNVFDVPSESEDDEEVEALKGEWSIGAELYYGIRERSGGVSAGIRYRTLPQFSSQSPLSVTYLINPIMGHMSAAFAAQVSDDLALCPRFDFNMYSYESDLIIGAEWWQREKSDIECENQSNDDTVNKMVLQGEVNGIVKATVGTSRGVTLLWEGRYGKTLFSLGLIADLTSKISPIRSIGLQFQYFS

>tr|U9UFF5|U9UFF5_RHIID Uncharacterized protein (Fragment) OS=Rhizophagus irregularis (strain DAOM 181602 / DAOM 197198 / MUCL 43194) GN=GLOINDRAFT_95481 PE=4 SV=1

QQQRLREQARNDEIPLHLREDYKDYNIQIDDQTQYEPSNSEGEEVPLKQDTDAQIHIEVSYKGNMKMVICTELYMNYPSLMFMSLPVRLTITGFEFSATAVVAYLRNRVNFCFLEPKNPEESHLKEVYIESEIGDKEKQVLKNVGKLEKFIIDQLRKIIDEDFVFPSYHSIEL

>tr|U9TTI3|U9TTI3_RHIID Mitochondrial distribution and morphology protein 34 OS=Rhizophagus irregularis (strain DAOM 181602 / DAOM 197198 / MUCL 43194) GN=MDM34 PE=3 SV=1

MAFKFNWPDFTTEFVEQAKQLLTTALNKSNKPANIVDHIVVKDLNMGTKPPELEIMEIGELAVDKFRGIFKLIYSGDAHLTLQTKVQANPMNSNKSDVSMYTRRGILAADQPLVVPMLLRLSNLKLRGIIVLVVSKQKGITLVFKNDPLEKVDISSTFDSISSIQRFLQTEIEKRLRIMFQEDLPSIVHQLSLRKWLNCNKQKEETTFKQELLEKDHNYDHSPLHNLPENTPYDTMSMPDLRYHTPLSTPSSEFSSISPESSFYAEDGYSNMGDLDSLDGYPGCSTYSNFGDLFDRQKEEGLKAISQPNKDHYSDVNRPRVFHRQRFLVSPKPQRRGSLPTWLPSVQEQQYDVRKPLRRHIHSEVKHPLSPIITDHFTETVSPASSSNINKVTMANLQRHNQYQTAERTQSLGHQNSLFSSNTSDDVTYNVSLSDSHQHINHKSVSASTFSSNEHDVFLRQQEIVLQPSETSVAAKLATLMNSNHTISPYTRTLQHLTFRSFPHPANKTLTNKRKLPGKVVVKRNVLSFPSLTTGFEHCNNNVHDSNNNKD

>tr|U9UF16|U9UF16_RHIID Uncharacterized protein OS=Rhizophagus irregularis (strain DAOM 181602 / DAOM 197198 / MUCL 43194) GN=GLOINDRAFT_172771 PE=4 SV=1

MNMLGKLSQSSLATRILGTTPSLYCNGRIVCVNLLRQYATTTTKNTKNTKKNTKNTKTTKTTKSTKTKRGSDSKSTQKKKKEKENVYLIPQEPKKPSTAYTLFFRDFFYERHKKNPEDKVTEIARLAGNEWVKLSAEEKNKYTNQYKEKRDEFKTSHEAWLRSLTLDQIAQENKRRRMEMSKGRRKSKLKLLKHPDQPKRPKTPYIHFVVERMSDNAEKGAVRIKELASEWRQLSDEDKEPYKKRYQQSKSEYETDMRAFEEKYIVTN

>tr|U9UI83|U9UI83_RHIID Aconitate hydratase, mitochondrial OS=Rhizophagus irregularis (strain DAOM 181602 / DAOM 197198 / MUCL 43194) GN=GLOINDRAFT_343775 PE=3 SV=1

MIARQLSSFLKNAHLSKKFPVRNYAQVLAGDILNKKIPISNFEKDKYINYKRIQDNLTTVKKRLDRPLTLSEKILYSHLDDPHGQDIKRGVSYLRLRPDRVACQDATAQMALLQFMSAGMPEVAVPTTVHCDHLIEAQIGGEKDLDRAIKLNKEVYDFLASASAKYNIGFWRPGSGIIHQIILENYAFPGGLMIGTDSHTPNAGGLGMVAIGVGGADAVDVMANIPWELKCPNVIGVKLTGSISGWTAPKDVILKVAGILTVKGGTGAIVEYFGPGVESLSCTGMATICNMGAEIGATTSLFPSNKRMVDFLRATKREEIASFSESFAHNLRADEGAQYDQLIEINLSELEPHINGPFTPDLATPISKFKDEVVKNDWPAELKVGLIGSCTNSSYEDMSRAASLAKQAIDHGIKAKSTFTITPGSEQIRATIARDGQEKVLKDVGGIVLANACGPCIGQWDRQDVKKGEKNSIITSYNRNFTGRNDANPATHAFVASPDIVTAFVFAGDLRFNPLTDTLIDANGKPFKFTGPTGDELPVKGYDPGQDTYKEPPADRSSVKVEVDPKSNRLQLLQPFSKWNGKDFEEFPILIKVKGKCTTDHISMAGPWLKFRGHLDNISNNMLIGAINAENNTANSVKNIFTGEYDAVPNVARDYKSKNT

PWVVIGDDNYGEGSSREHAALEVRHLGGAAVIVKSFARIHETNLKKQGLLPLTFANPSDYDKIDPLDKVSIVGLTTFAPGKPLTLRVHKKTGETVDITVKHTFNENQIEWFKAGSALNRMAEKHQN

>tr|U9UJR1|U9UJR1_RHIID Uncharacterized protein OS=Rhizophagus irregularis (strain DAOM 181602 / DAOM 197198 / MUCL 43194) GN=GLOINDRAFT_343557 PE=4 SV=1

MNFTRFFFRSLSRSSNAAFLKQQRHVIVGSKSYSILKSSTPRKAVSVKVNTLNDLKHSHSNQIRTMKTLDFGGTKEVVYERSDWPKEKFQEFFKNDTIAVIGYGPQGSGQSLNARDNGLNIIIGLREGTIIFNLLSDAAQKELWPKFVPHLTKGKTLYFSHGFSIVFKDETHVIPPKDIDVIMVAPKGSGRTVRSLFLDKRGINSSIAIFQDVSGKAKDTAVALGVAIGSGYLYETTFEREVFSDLFGERGVLMGAIQGLFLAQYEVLRSRGHTPSEAFNETVEEATQSLYPLIGANGMDWMYAACSTTAQRGALDWYKPFRDANKPVFERLYEAVANGSETRRVLEKNSSPTYREELEKELEEIRESEIWKTGKTVRNLRPENK
